# Characteristics of the Life Cycle of Porcine Deltacoronavirus (PDCoV) In Vitro: Replication Kinetics, Cellular Ultrastructure and Virion Morphology, and Evidence of Inducing Autophagy

**DOI:** 10.3390/v11050455

**Published:** 2019-05-18

**Authors:** Pan Qin, En-Zhong Du, Wen-Ting Luo, Yong-Le Yang, Yu-Qi Zhang, Bin Wang, Yao-Wei Huang

**Affiliations:** 1 Institute of Preventive Veterinary Medicine and Key Laboratory of Animal Virology of Ministry of Agriculture, College of Animal Sciences, Zhejiang University, Hangzhou 310058, China; qinpan@zju.edu.cn (P.Q.); duenzhong519@126.com (E.-Z.D.); 21617039@zju.edu.cn (W.-T.L.); yang-yl@zju.edu.cn (Y.-L.Y.); 3160100223@zju.edu.cn (Y.-Q.Z.); wb1990boy@163.com (B.W.); 2 YEBIO Bioengineering Co., Ltd. of Qingdao, Qingdao 266114, China

**Keywords:** Porcine deltacoronavirus (PDCoV), electron microscopy, ultrastructure, autophagy

## Abstract

Porcine deltacoronavirus (PDCoV) causes severe diarrhea and vomiting in affected piglets. The aim of this study was to establish the basic, in vitro characteristics of the life cycle such as replication kinetics, cellular ultrastructure, virion morphology, and induction of autophagy of PDCoV. Time-course analysis of viral subgenomic and genomic RNA loads and infectious titers indicated that one replication cycle of PDCoV takes 5 to 6 h. Electron microscopy showed that PDCoV infection induced the membrane rearrangements with double-membrane vesicles and large virion-containing vacuoles. The convoluted membranes structures described in alpha- and beta-coronavirus were not observed. PDCoV infection also increased the number of autophagosome-like vesicles in the cytoplasm of cells, and the autophagy response was detected by LC3 I/II and p62 Western blot analysis. For the first time, this study presents the picture of the PDCoV infection cycle, which is crucial to help elucidate the molecular mechanism of deltacoronavirus replication.

## 1. Introduction

Porcine deltacoronavirus (PDCoV), which belongs to the genus *Deltacoronavirus* in the subfamily Coronavirinae of the family Coronaviridae, order Nidovirales, was first reported in Hong Kong in 2012 [1] and thereafter isolated from pigs in the United States [2] and many Asian countries including China [3,4,5]. It causes acute diarrhea, vomiting, dehydration and mortality in nursing pigs [6,7]. The PDCoV genome is a positive sense single-stranded RNA of approximately 25.4 kb in size. The genome organization of PDCoV is similar to those of other reported coronaviruses, with the typical gene order 5′-ORF1a/1b-Spike (S)-Envelope (E)-Membrane (M)-NS6-Nucleocapsid (N)/NS7-3′. Ultrastructural characterization of other CoVs in alpha-, beta-, or gamma-CoV genus such as severe acute respiratory syndrome coronavirus (SARS-CoV) [8,9,10,11,12,13,14], human coronavirus NL63 (HCoV-NL63) [15], infectious bronchitis virus (IBV) [16], transmissible gastroenteritis virus (TGEV) [17] and porcine epidemic diarrhea virus (PEDV) [18] has been performed. The studies on three CoV genera demonstrated that the architectures of the organelle during CoV infection are distinct between among alpha-/beta-coronaviruses and gammacoronavirus. Alpha- and beta-coronaviruses formed clusters of the double-membrane vesicle (DMV), which is highly conserved among coronaviruses, sometimes linked by a convoluted membrane [11,15,19,20,21], whereas the gammacoronavirus IBV induced extensive paired membranes and smaller 60-80 nm spherules in addition to the DMVs [16].

There is paucity of information on the ultrastructural picture of the newly discovered deltacoronavirus infection. The isolation, epidemiology, and pathogenicity of PDCoV has been recently studied by researchers from different countries, but a better understanding of the PDCoV life cycle is critically needed to help elucidate the mechanism of virus replication and antiviral activity of the host cells. In this study, some basic, in vitro characteristics of the life cycle, for example replication kinetics, cellular ultrastructure, virion morphology, and possible induction of autophagy of PDCoV, were studied and elaborated.

## 2. Materials and Methods

### 2.1. Cell and Virus

The PDCoV Chinese “Hunan” strain was used in this study [22]. The virus cultivation was done in a porcine kidney epithelial cell line, LLC-PK1 (ATCC CL-101), at 37 °C in 5% CO2 in Dulbecco’s modified Eagle’s medium (DMEM) supplemented with 10% fetal bovine serum (FBS) and 1% (*w*/*v*) antibiotics (penicillin and streptomycin). 

### 2.2. Extracellular and Intracellular Viral RNA Quantitation

LLC-PK1 cells were seeded at 80% confluence in 6-well culture plates and incubated overnight. After the cells were washed with phosphate-buffered saline (PBS), viruses at a multiplicity of infection (MOI) of 1 were added to each plate. After 1 h of incubation at 37 °C, the cells were washed four times with PBS and then harvested along with supernatants at an hourly interval until 12 h post-infection (hpi). At least three replicate experiments were performed. Viral subgenomic RNA (sgRNA) load was monitored by one-step quantitative reverse-transcription polymerase chain reaction (qRT-PCR) targeting the membrane (M) gene as described previously (forward primer, 5′-ATCGACCACATGGCTCCAA-3′; reverse primer, 5′-CAGCTCTTGCCCATGTAGCTT-3′; and probe, 5′-FAM-CACACCAGTCGTTAAGCATGGCAAGCT-BHQ-3′ [23], as the M gene is expressed by the sgRNA, and viral genomic RNA (gRNA) load was detected by qRT-PCR targeting the non-structural protein (nsp) 2 encoding region (forward primer, 5′-GAAGGTGAAGATGATAGTG-3′; reverse primer, 5′-GCTCTGGTTTAGGATAGA-3′; and probes, 5′-FAM-TACTTCGTCGCTGCTGGTCTT-TAMRA-3′, as the nsp2 of ORF1a is expressed by the gRNA. Standard curves were performed to allow absolute quantitation of PDCoV RNA copy numbers based upon the levels of in vitro transcribed RNAs containing the targeting sequences [24].

### 2.3. Virus Yield Assay

LLC-PK1 cells were infected with PDCoV at an MOI of 5 and cell supernatants were harvested hourly, to determine the onset of progeny virus release from the infected cells. The titers of the yield of progeny virus were determined by endpoint dilutions as 50% tissue culture infective dose (TCID_50_) on LLC-PK1 cells.

### 2.4. Transmission Electron Microscopy (TEM)

LLC-PK1 cells were infected with PDCoV at an MOI of 1. The cells were harvested and fixed with 2.5% glutaraldehyde in phosphate buffer (0.1 M, pH 7.0) and 1% Osmium tetroxide (OsO4) in phosphate at 2, 4, 6, 8, 12, 16, and 24 hpi. Specimens were dehydrated in a series of ethanol dilutions (30%, 50%, 70%, 80%, 90%, 95% and 100%) for 15–20 min at each step, then transferred to absolute acetone for 20 min. The specimens were then placed in one of three mixtures of absolute acetone and Spurr resin (1:1, 1:3, and pure Spurr resin) for 1 h, 3 h, and overnight, respectively. Finally, ultrathin sections were stained by uranyl acetate and alkaline lead citrate for 5–10 min and observed using a Hitachi Model H-7650 transmission EM [25].

### 2.5. Western Blot Analysis

PDCoV infected LLC-PK1 cells were lysed in CelLytic M lysis buffer (Sigma, St. Louis, MO, USA). The protein concentration was quantified by the BCA (bicinchoninic acid) protein assay kit (Beyotime Biotechnology, Shanghai, China). Samples were separated by 12% sodium dodecyl sulfate-polyacrylamide gel electrophoresis (SDS-PAGE). The proteins were transferred onto a polyvinylidene difluoride (PVDF) membrane that was subsequently blocked with Tris-buffered saline (TBS) containing 3% bovine serum albumin (BSA) at room temperature for 2 h and then incubated overnight at 4 °C with primary antibodies. Rabbit anti-LC3 (A7198, ABclonal, Wuhan, China) and rabbit anti-p62/SQSTM1 (P0067, Sigma, St. Louis, MO, USA) polyclonal antibodies were used in this study. The blots were then incubated with corresponding horseradish peroxidase (HRP) conjugated secondary antibody (Thermo Fisher Scientific, Waltham, MA, USA).

## 3. Results and Discussion

### 3.1. In Vitro Porcine Deltacoronavirus (PDCoV) Replication Kinetics

**Figure 1:**
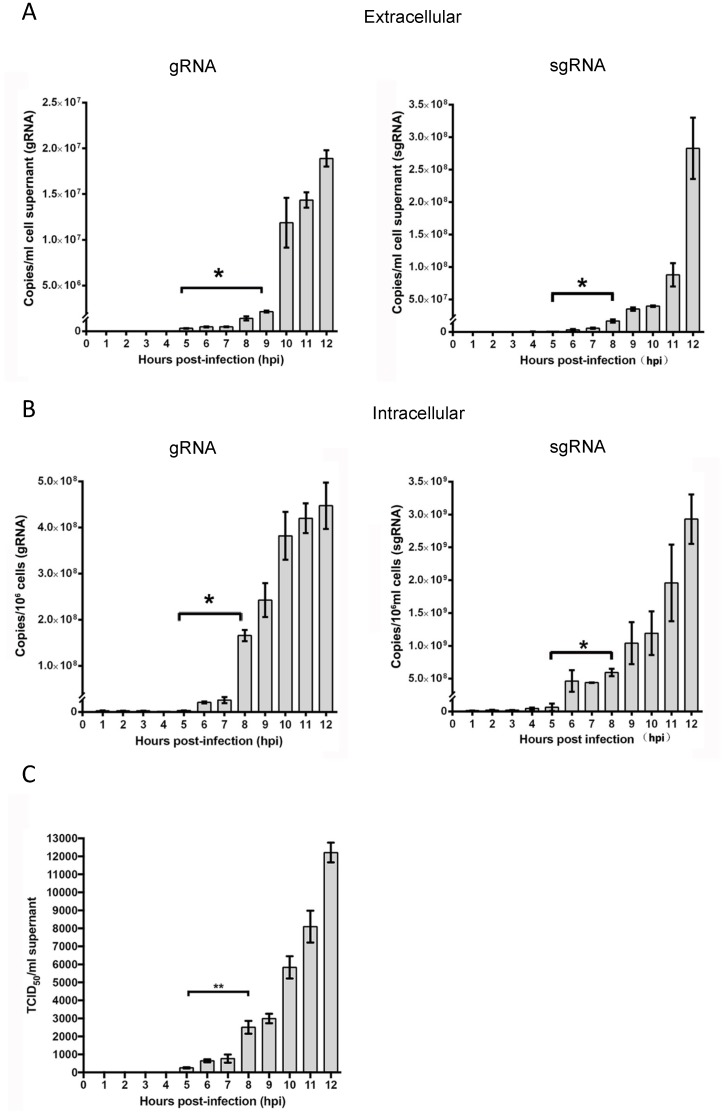
Determination of the growth kinetics and the time of one life cycle of porcine deltacoronavirus (PDCoV) infection in porcine kidney epithelial (LLC-PK1) cells. The amounts of extracellular gRNA and sgRNA (**A**), as well as intracellular gRNA and sgRNA (**B**) were determined by quantitative reverse-transcription polymerase chain reaction (qRT-PCR), respectively. Samples of supernatants and cells were collected at hourly intervals between 1 to 12 h post-infection. Viral titers were determined by end-point dilutions on fresh LLC-PK1 cells and calculated as TCID_50_ per mL (**C**). Data represent mean values ± standard deviation (SD) of at least 3 replicates. Differences in the RNA copies or virus titer were evaluated by *t*-test, Asterisks indicate significant differences between the two groups, * *p* < 0.05; ** *p* < 0.01.

Viral gRNA was first detected in the supernatants at 5 hpi, which increased significantly at 9 hpi (*p* < 0.01) and reached a maximum of 1.88 × 10^7^ copies/mL at 12 hpi (Figure 1A, left panel). Intracellular gRNA load remained low until 5 hpi, followed by a sharp increase at 6 hpi from 2.92 × 10^6^ to 2.07 × 10^7^ copies/10^6^ cells, reaching a final titer of 4.47 × 10^8^ copies/10^6^ cells at 12 hpi (Figure 1B, left panel). Similarly, extracellular or intracellular viral sgRNA amount gradually increased from 5 hpi, with significant changes at 9 or 8 hpi (Figure 1A,B, right panels). Infectious progeny virus could be detected in the culture supernatants at 6 hpi at a low titer (4.5 × 10^2^ TCID_50_/mL), and then the amounts of progeny virus increased. By 12 h post infection, infected LLC-PK1 cells produced an infectious titer of 1.25 × 10^5^ TCID_50_/mL (Figure 1C). These results indicated that the first generation of progeny was assembled and released from infected cells at about 5 hpi, suggesting that one replication cycle of PDCoV takes approximately 5 to 6 h to complete.

### 3.2. PDCoV Infection Induces Cellular Ultrastructural Changes

**Figure 2:**
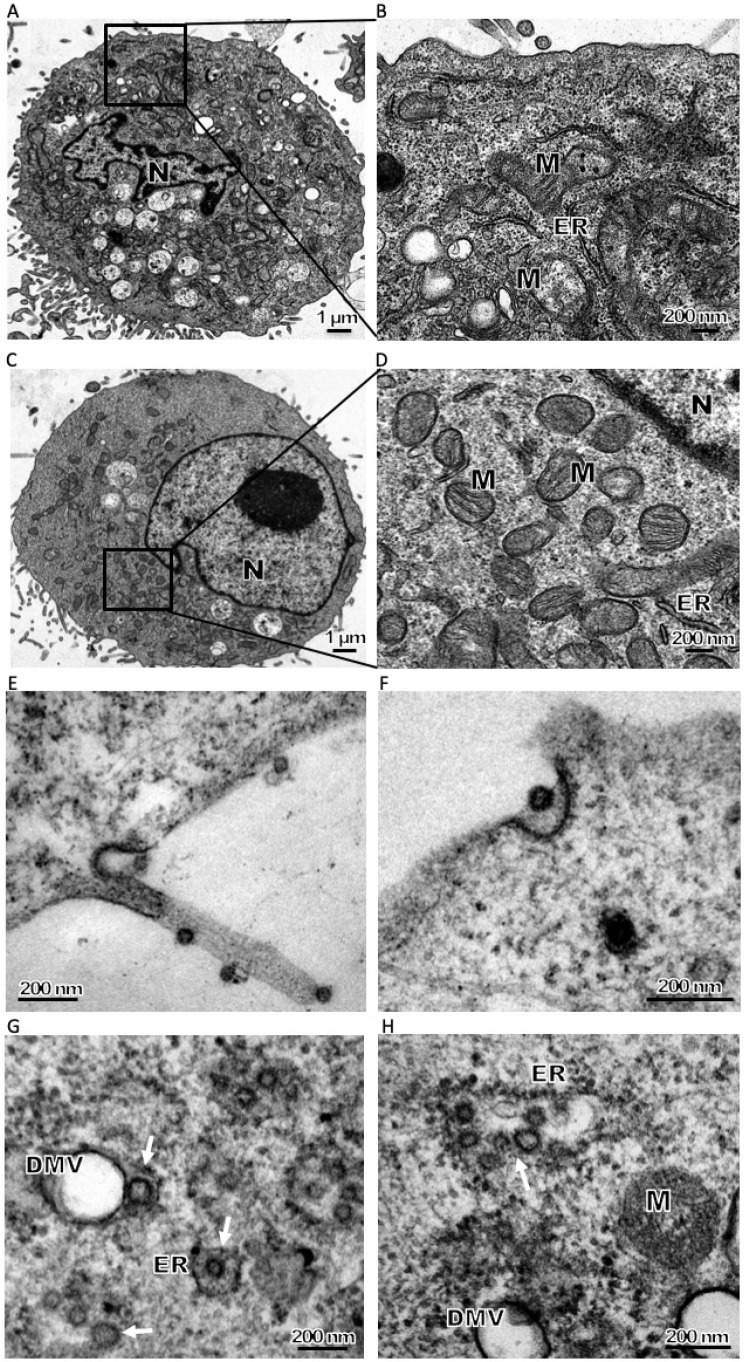

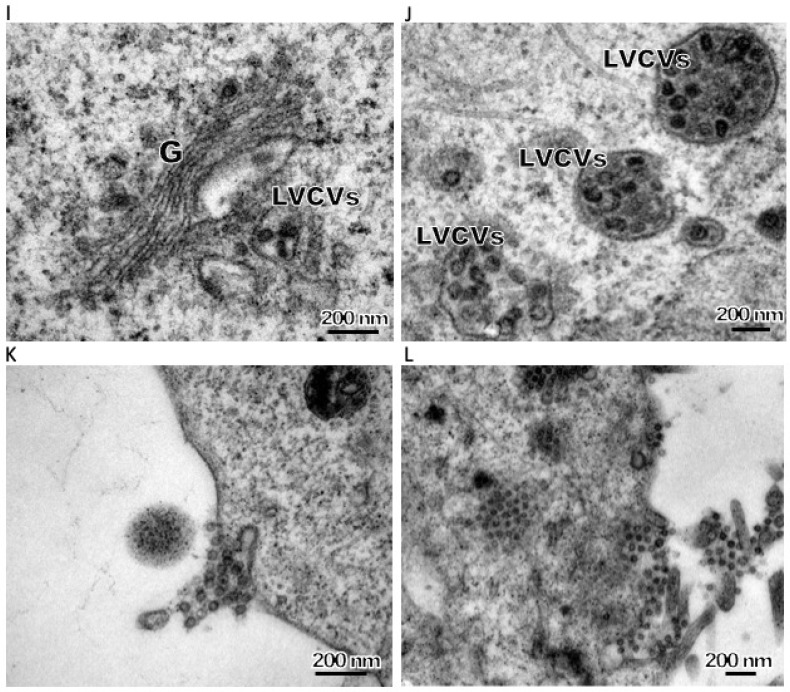
Electron microscopy (EM) observation of in vitro PDCoV infection: entry, assembly and maturation of PDCoV particles. LLC-PK1 were infected (**A**,**B**) with PDCoV or mock-infected (**C**,**D**) at 8 h post-infection (hpi). (**B**,**D**) Zoomed region highlighted in Figure A and C respectively. Compared with mock-infected cells large amounts of vesicles, including double-membrane vesicles (DMVs), zippered endoplasmic reticulum (ER) and injured mitochondria, are visible in the cytoplasm. (**E**) Viral particles in the process of attachment on the cell membrane at 4 hpi, and one viral particle likely in the process of endocytosis at 4 hpi. (**F**) One viral particle is likely in the process of endocytosis at 4 hpi. (**G**) Precursor PDCoV virions assembled predominantly in the rough endoplasmic reticulum (RER) nearby DMVs (white arrow, hpi = 6). (**H**) Precursor virions assembled in the RER or ERGIC. Precursor particles were observed in the dilated RER (white arrow) (hpi = 8). (**I**) Precursor virions likely completed the structural transformation that yielded small-size virions. Large virion-containing vacuoles (LVCVs) are thought to originate from Golgi compartments that expand to accommodate numerous virions (hpi = 16). (**J**) LVCVs are large circular organelles with a diameter of approximately 400–600 nm that contain numerous virions in their interior (hpi = 16). (**K**) Virion-containing vesicles fuse with plasma membranes and then released the virus particles to the extracellular environment without damage to the cell in the first few replicative cycles (hpi = 12). (**L**) Large amounts of viral particles released by cytolysis (hpi = 24). ER: endoplasmic reticulum, M: mitochondria, N: nucleus, G: golgi apparatus.

The diameter of known CoV particles ranges from 70–120 nm. The size of the purified PDCoV virion by ultracentrifugation (the approach described in [25]) was first assessed. Electron microscopy (EM) of a negatively stained sample demonstrated that the virus particle was 58 to 70 nm in diameter, and had surface projections typical of CoV (Supplemental Figure S1). PDCoV infected cells exhibited significant morphologic changes (Figure 2A,B) compared to uninfected controls (Figure 2C,D) at 8 hpi, such as the presence of many cytoplasmic vesicles including dilated rough endoplasmic reticulum, DMVs and injured mitochondria. The cytoplasmic vesicles surrounding the nucleus increased along with infection (Figure 2A,B). The size of PDCoV virions observed with infected cells, approximately 50–70 nm in diameter (Figure 2E,F), was in line with the purified viral particle (Supplemental Figure S1). 

CoV infection is initiated by the interaction of the viral S protein with specific host cellular receptors followed by membrane fusion, and the cellular receptor for PDCoV entry has just been identified as porcine aminopeptidase N [24]. At 4 hpi, PDCoV virions were seen attached to the cell surface (Figure 2E). The process of likely endocytosis of PDCoV was also observed (Figure 2F), which needs further confirmation. Following internalization, CoV particles are transported and scattered into the cytoplasm. It is well known that viral gRNA is recognized directly by the host cell machinery and translated into non-structural proteins, which assemble into viral replication–transcription complexes (RTCs) on DMVs or other membrane structures [16,21,26], coordinating the enzymes required for viral RNA transcription, proof-reading and capping of new viral transcripts. Similar events should apply to PDCoV infection as a large number of DMVs were seen at 6 hpi and thereafter. After completion of genome replication and RNA transcription, the virions of PDCoV start to assemble in the expanded rough endoplasmic reticulum (RER) with irregular shape, as the surface was decorated with ribosomes (Figure 2G,H). Immature PDCoV precursors with an electron-dense periphery and clear core were present in the RER (Figure 2H) or in the lumen of a stack of adjacent cisternae, very likely the Golgi apparatus (Figure 2I). The precursors likely trafficked through the endoplasmic reticulum–Golgi intermediate compartment (ERGIC), completing structural transformation into small, infectious virions in the Golgi complex. The virions were also observed in large circular organelles (Figure 2J). These structures, named as Large virion-containing vacuoles (LVCVs), have earlier been described for other CoVs-infected cells, which are thought to originate from Golgi compartments that expand to accommodate numerous virions [27]. Mature virions accumulate inside secretory vesicles, which tend to merge into bigger vesicles (Figure 2J). After that, vesicles containing mature virions moved to the cell periphery and eventually fuse with the cell membrane. In the final phase of infection, mature virions must escape the cell. PDCoV release was observed to occur via two pathways: exocytosis and cytolysis. Early virions, those generated within the first few replicative cycles, may be released by exocytotic fusion of virus-containing vesicles with plasma membranes without damage to the cell (Figure 2K). However, as viral infection progressed and the quantity of virus in the cells increased, large amounts of virus particles were released by cytolysis resulting in necrosis of the host cell (Figure 2L). 

**Figure 3:**
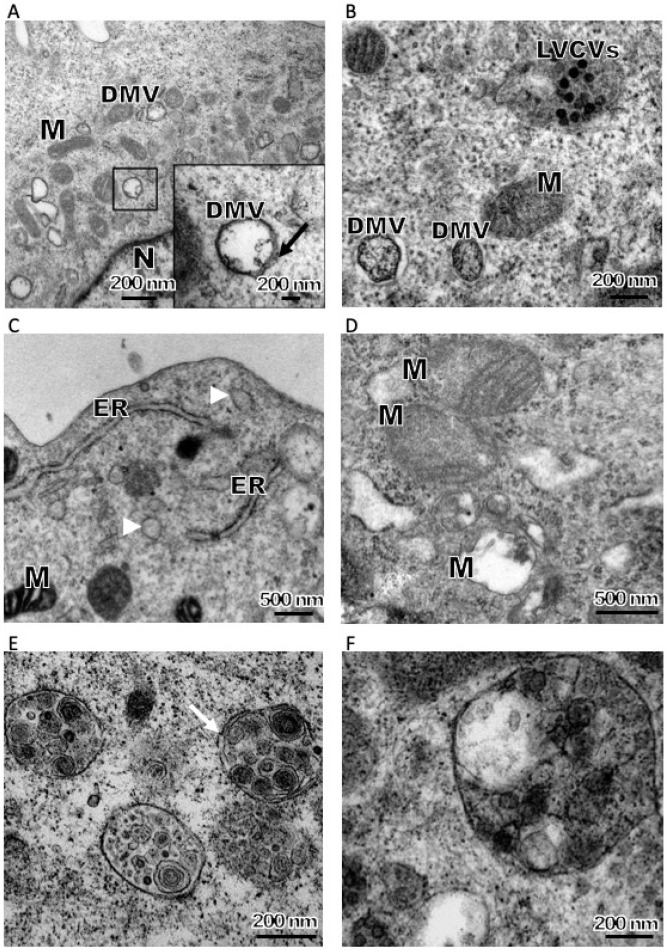
Representative images from EM of PDCoV induces membrane structures. (**A**,**B**) Double membrane vesicles (DMVs) induced by PDCoV infection. Most of the DMVs, lacking the fibrous material, were separate entities or in small clusters in the cytoplasm, and the inner and outer membranes were generally tightly apposed (**A**). DMVs containing an electron-dense core and luminal spacing between the outer and inner membranes also could be observed (Ainset). DMVs containing an electron-dense core also could be observed (**B**). (**C**) PDCoV infection induces the zippered ER and the ER curved to form a double-membrane vase-like compartment (white arrowhead). (**D**) Upon PDCoV infection, damages of the mitochondria, showing the disappearance of cristae, could be observed. (**E**,**F**) PDCoV infection induced autophagosome-like compartments in the cytoplasm of infected LLC-PK1 cells. Autophagosome-like vacuoles or lysosomes contain RER, small vesicles, mitochondrion and other vesicles. Double membrane of the vacuoles is partially visible (arrow). ER: endoplasmic reticulum, M: mitochondria, N: nucleus.

CoV replication is closely tied to the formation of membrane-bound RTCs that alter cell membrane structures, as previously reviewed [19,28]. Many such alterations were observed directly in an electron microscope (EM) of PDCoV-infected LLC-PK1 cells, including DMVs (Figure 2G,H and Figure 3A,B), LVCVs (Figure 2I,J), zippered ER (Figure 3C) and damaged mitochondria (Figure 3D). The DMV architecture is a typical and highly conserved feature of CoV infections, with a diameter ranging from 150–300 nm with electron-dense cores [8,9,11,13,14,15,16,26,29]. DMVs play a role not only in the synthesis of viral RNA [21] but also in helping to concentrate on viral proteins and offer protection from cellular antiviral detection and elimination machinery [28]. DMVs could be detected at 2 hpi in SARS-CoV or mouse hepatitis virus (MHV) infected cells and then the number of DMVs increased dramatically [11,27]. The appearance of DMVs in PDCoV-infected cells was seen at 6 hpi (Figure 2G). The sizes of DMVs ranged from 200 to 400 nm. PDCoV induced a low number of DMVs and most of the DMVs were separate entities or in small clusters in the cytoplasm. Initially, the DMV inner and outer membranes were generally tightly apposed (Figure 2G,H) but, occasionally, some luminal space between the two lipid bilayers could be discerned (Figure 3A). Similar observations were previously made for SARS-CoV and IBV [11]. Most of the DMVs contained few or no fibrous material in the inner vesicles (Figure 2G,H and Figure 3A). Interestingly, DMVs containing an electron-dense core could also be observed (Figure 3B). Previous researches confirmed that the detection of this core depended on the protocol used for the preparing of resin-embedded specimens. In chemically fixed samples, the interior of DMV appeared mainly electron translucent. However, the core can be preserved by using high-pressure freezing followed by freeze substitution. A similar phenomenon can be observed in the IBV infected cells and chemically fixed tracheal organ cultures. Two types of DMVs could well represent early and later stages in the existence of a DMV [30]. Previous research suggested that CoV DMV structures derive from ER membranes [11,12,13], though the precise mechanism of membrane rearrangement remains controversial. In the context of nidovirus-induced DMV formation, two alternatives have been proposed: enwrapping and double budding [30]. The multiple EM images of zippered ER and small vesicles close to the zippered ER in PDCoV infected cells (Figure 3C) suggest that these structures were likely DMV precursors. 

Interestingly, convoluted membranes structures or spherules, which can be observed in proximity to the DMV cluster in alpha- or gamma coronavirus-infected cells, were not detected in the PDCoV infected cells, despite analysis at several time points post-infection (Figure 2 and Figure 3). The large evolutionary distance between the coronavirus genera might be attributed to such differences. Previous studies proposed that these structures were the site of viral genome translation and polyprotein processing. Further work will be needed to determine the details on membrane structures that participate in PDCoV RNA synthesis. PDCoV infection was shown to induce mitochondrion damage. Irregular mitochondria and mitochondria lacking cristae were observed after PDCoV infection, indicative of injured mitochondria (Figure 3D). We hypothesize that these structures may donate their membrane material to form autophagosomes [31], as described below. 

### 3.3. PDCoV Infection Induces Autophagosome-Like Vesicles Associated with Increased Autophagic Activity

Autophagy is a dynamic and continuous cellular defence pathway, resisting viral infection by degrading both virus and necrotic organelles. Regions of the cytoplasm become engulfed into large double membrane-bound vesicles termed autophagosomes, which are another type of distinct structures seen during CoV infection [29,31]. Autophagosomes are distinguished from DMVs by the size and specific markers of autophagic activity. Double-membraned autophagosomes-like vesicles were detected in PDCoV-infected cells at 12 hpi and increased thereafter. The initial autophagic vacuoles contain cytosol and/or organelles that in appearance are morphologically intact, and similar to the cytosol and organelles elsewhere in the cell. The membrane of the vacuoles is partially visible as two bilayers separated by a narrow electron-lucent cleft [32] (Figure 3E,F). It should be noted that these are characteristic features of coronavirus infection, and expression of SARS-CoV nsp3 alone induced similar structures.

**Figure 4:**
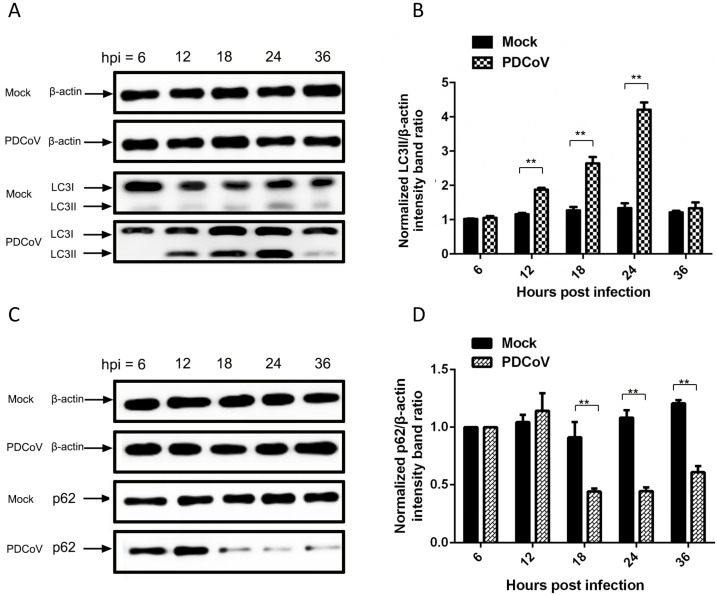
PDCoV infection increased the conversion of LC3-I to LC3-II (**A**,**B**) as well as degradation of p62 proteins in LLC-PK1 cells (**C**,**D**). LLC-PK1 cells were mock-treated or infected with PDCoV (multiplicity of infection (MOI) = 1). At 6, 12, 18, 24 and 36 hpi, cells were lysed and subjected to western blot with antibodies against LC3, p62 and β-actin (as the loading control). The protein blots were quantified by the Image J software. The ratio of LC3-II to β-actin and the ratio of p62 to β-actin from three independent experiments were expressed as mean ± standard deviation (SD); ** *p* < 0.01, calculated using Student’s *t*-test.

Upon induction of autophagy, a series of conjugation reactions converts cytosolic microtubule-associated protein light chain 3 (LC3-I) to a lipidated form (LC3-II); the ratio of LC3-II/LC3-I is regarded as an accurate indicator of autophagic activity [33]. In order to further confirm that PDCoV induced autophagy, LC3 conversion in PDCoV-infected LLC-PK1 cells was determined by Western blot using an antibody recognizing both forms of LC3. The LC3-II/LC3-I ratio increased significantly from 12–24 hpi in PDCoV-infected cells relative to mock-infected cells (Figure 4A,B). This was consistent with the increase in autophagosomes observed in infected cells from 12–18 hpi under EM (Figure 3E,F). We also monitored autophagic flux, using western blot assay to measure changes in p62 protein levels upon infection. Because p62-bound polyubiquitinated proteins become incorporated into the completed autophagosome and are degraded in autolysosomes, they serve as an index of autophagic degradation [34]. PDCoV infection increased degradation of p62 in LLC-PK1 cells starting at 18 hpi (Figure 4C,D), indicating that accumulation of autophagosome-like vesicles is linked to autophagic activation. These results provided evidence that PDCoV infection may induce autophagy just like other CoVs [29,35,36,37].

A general response to viral infections is the activation of autophagy, which either has a positive or negative outcome depending on the nature of the virus. Initial work with MHV showed that although capable of inducing autophagy, it was replication-deficiet in ATG5^−/−^ embryonic stem cell lines (lacking ATG5, the E3 ubiquitin ligase essential for autophagosome elongation). This replication deficiency reversed in the presence of an ATG5-expressing plasmid [29]. However, other studies showed that MHV and SARS-CoV replication does not require an intact autophagy pathway [38,39]. Similar results were also observed in IBV infection, where induction or inhibition of autophagy did not affect replication, suggesting that classical autophagy may not be important for its life cycle [40]. However, a recent study shows that TGEV infection activates autophagy, which subsequently inhibits further TGEV infection [36]. As it is possible that replication mechanisms differ among viruses of the same family, further in-depth study is needed to confirm PDCoV infection-associated autophagy and identify the impact of autophagy on viral replication.

## 4. Conclusions

In summary, we demonstrate and elaborate some novel, in vitro characteristics of the life cycle of PDCoV, including replication kinetics in cultured cells, cellular ultrastructure, virion morphology, and possible induction of autophagy, mainly by means of EM observation. PDCoV infection induced membrane rearrangements. However, convoluted membrane structures, caused by α-CoV, were not observed in the PDCoV-infected cells. This study gives the first detailed picture of the PDCoV infection cycle, which will help elucidate the molecular mechanism of deltacoronavirus replication.

## Supplementary Materials

The following are available online at https://www.mdpi.com/1999-4915/11/5/455/s1. Figure S1. Electron microscope image of a purified PDCoV particle using phosphotungstic acid negative staining.
