# Supplementary material for: Characteristics of the Life Cycle of Porcine Deltacoronavirus (PDCoV) In Vitro: Replication Kinetics, Cellular Ultrastructure and Virion Morphology, and Evidence of Inducing Autophagy
Source: Viruses. 2019 May 18;11(5):455. doi: 10.3390/v11050455 (PMC6563515; doi:10.3390/v11050455)
Supplement: Supplementary file 1 [file viruses-11-00455-s001.pptx]

## Slide 1
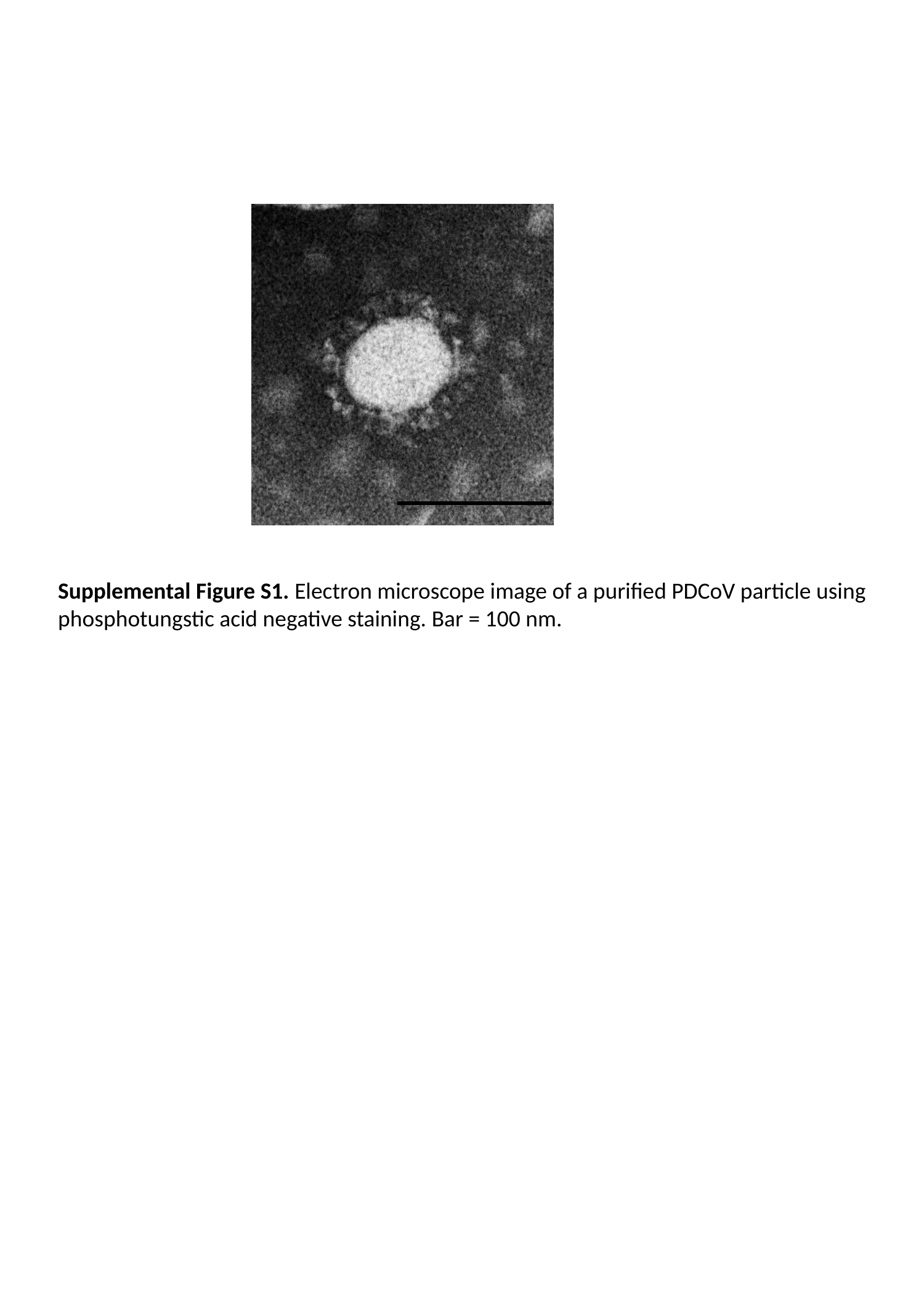

Supplemental Figure S1. Electron microscope image of a purified PDCoV particle using phosphotungstic acid negative staining. Bar = 100 nm.
